# The factor structure and psychometric properties of the Clinical Outcomes in Routine Evaluation – Outcome Measure (CORE-OM) in Norwegian clinical and non-clinical samples

**DOI:** 10.1186/1471-244X-13-99

**Published:** 2013-03-22

**Authors:** Ingunn Skre, Oddgeir Friborg, Sigmund Elgarøy, Chris Evans, Lars Henrik Myklebust, Kjersti Lillevoll, Knut Sørgaard, Vidje Hansen

**Affiliations:** 1 Department of psychology, University of Tromsø, Tromsø, Norway; 2 Sami national competence centre for mental health services (SANKS), Finnmark health trust, Lakselv, Norway; 3 Nottinghamshire healthcare NHS trust, Nottingham, UK; 4 Centre for psychiatric research, University hospital of North Norway, Tromsø, Norway; 5 Psychiatric research centre of North Norway, Nordland hospital trust, Bodø, Norway; 6 Department of clinical medicine, University of Tromsø, Tromsø, Norway

**Keywords:** Outcome measure, CORE-OM, Translation, Reliability, Confirmatory factor analysis

## Abstract

**Background:**

The Clinical Outcomes in Routine Evaluation - Outcome Measure (CORE-OM) is a 34-item instrument developed to monitor clinically significant change in out-patients. The CORE-OM covers four domains: well-being, problems/symptoms, functioning and risk, and sums up in two total scores: the mean of All items, and the mean of All non-risk items. The aim of this study was to examine the psychometric properties of the Norwegian translation of the CORE-OM.

**Methods:**

A clinical sample of 527 out-patients from North Norwegian specialist psychiatric services, and a non-clinical sample of 464 persons were obtained. The non-clinical sample was a convenience sample consisting of friends and family of health personnel, and of students of medicine and clinical psychology. Students also reported psychological stress. Exploratory factor analysis (EFA) was employed in half the clinical sample. Confirmatory (CFA) factor analyses modelling the theoretical sub-domains were performed in the remaining half of the clinical sample. Internal consistency, means, and gender and age differences were studied by comparing the clinical and non-clinical samples. Stability, effect of language (Norwegian versus English), and of psychological stress was studied in the sub-sample of students. Finally, cut-off scores were calculated, and distributions of scores were compared between clinical and non-clinical samples, and between students reporting stress or no stress.

**Results:**

The results indicate that the CORE-OM both measures general (*g*) psychological distress and sub-domains, of which risk of harm separates most clearly from the *g* factor. Internal consistency, stability and cut-off scores compared well with the original English version. No, or only negligible, language effects were found. Gender differences were only found for the well-being domain in the non-clinical sample and for the risk domain in the clinical sample. Current patient status explained differences between clinical and non-clinical samples, also when gender and age were controlled for. Students reporting psychological distress during last week scored significantly higher than students reporting no stress. These results further validate the recommended cut-off point of 1 between clinical and non-clinical populations.

**Conclusions:**

The CORE-OM in Norwegian has psychometric properties at the same level as the English original, and could be recommended for general clinical use. A cut-off point of 1 is recommended for both genders.

## Background

Valid instruments for systematic routine outcome measurement may be key tools for preventing treatment failure in psychotherapy [1,2]. The clinical value of outcome-scores gained through translated instruments depends upon that both content and test-parameters are comparable to the original language version, that the scale scores are sensitive to change in subjective psychological stress, and finally, upon whether the tool separates a clinical sample from a non-clinical sample [3].

The Clinical Outcomes in Routine Evaluation-Outcome measure (CORE-OM) was developed to track the status of patient mental health problems through the course of outpatient treatment [4,5]. Items were carefully chosen according to their clinical significance and to their likeliness to change during recovery. The items cover four domains: well-being, problems/symptoms, functioning and risk. *Well-being* depicts the affective tone, and the quality of life of the patient. *Problems/symptoms* include the most common symptoms of anxiety and depression, aftermath of trauma, and physical correlates of psychological health. *Functioning* indicates functioning in daily life, in general, as well as in social and close relations. *Risk* covers self-harm and suicidal ideation, as well as threats of violence, and perpetrated violence against others. The first three domains correspond with the phase model for psychotherapy change [6], which entails progressive improvement, firstly by improved well-being, secondly by reduction in symptoms, and, finally, by enhancement of life functioning. The fourth domain, risk, was chosen to assist the clinician in monitoring the most adverse outcome, namely signs of risk of suicide, self-harm and violence in patients with mental health problems.

The three first CORE domains (well-being, problems and functioning), have been found to be highly correlated, while the risk domain shows more moderate correlations with the other domains [7-9]. Two studies of the factor structure of the original English CORE-OM have not confirmed the theoretical model of four independent domains [9,10]. Lyne and colleagues [9] found evidence that the 34 items shared a *g* (general) factor of psychological distress, with residual wellbeing, problems, functioning, risk factors, and finally, separate factors for positively and negatively worded items. They did however find evidence that the risk items separated into “risk to self” and “risk to others”, and together formed a separate factor, important for clinical flagging of risk of harm. They also looked for gender differences in factor structure, and found that although present, gender differences were of little clinical significance. Consequently, Lyne and colleagues [9] concluded with a recommendation for using CORE-OM as a two-scale instrument: one general scale measuring psychological distress, and containing all non-risk items, and one scale containing the risk items. In the same line, using principal component analyses (PCA) and Mokken scaling, Bedford and colleagues [10] demonstrated a two-factor structure of the CORE-OM, and argued for shortening the scale. The high internal consistency of the distress factor clearly indicated that the CORE-OM contained redundant items. Bedford and colleagues [10] raised the question whether the CORE not only measured the state during the last week, but also touched upon stable traits, in other words that the CORE also measured personality factors [11].

The Norwegian translation of CORE-OM was conducted in 2001, and was the first approved translation of the CORE-OM. At present approved translations of the CORE-OM exist in most European languages. Scientific validations have hitherto been published of the Italian [12], the Swedish [7] and Japanese translations [13]. The test-retest stability of the CORE-OM has been found acceptable in the Swedish [7], the English [8] and the Japanese [13] language versions. Convergent validity of the CORE-OM towards measures of anxiety and depression has also been found acceptable [7,8,12,13]. Elfström and colleagues [7] also demonstrated through a language experiment, presenting alternate Swedish and English versions of the CORE-OM to a student sample, that language did not influence the scores, thereby further validating the content of the Swedish version.

Sensitivity to change in symptomatology and stress is important for an outcome measure. In the British validation of the CORE-OM, studying cut-off points for clinically significant change, Evans and colleagues [8] found that females had higher or equal scores compared to men on all scales, except risk, for which men scored higher. The total score cut-off point was 1.2 for men and 1.3 for women. In a more recent study Connell and colleagues [14] argued for rounding the clinical cut-off score down to 1.0 for both sexes by demonstrating that this clinical cut-off separates between patients and non-distressed asymptomatic individuals from the general population. The Swedish validation [7] of CORE-OM also demonstrated that CORE is sensitive to change in a patient sample during out-patient treatment.

The aims of this paper were to study the psychometric properties of the Norwegian version of the CORE-OM:

(i) by exploring and confirming the factor structure  of the Norwegian CORE-OM;

(ii) by exploring the internal consistency and test-retest  stability;

(iii) by studying the influence of current patient status, gender and age;

(iv) by calculating cut-off points for clinical significant  change;

(v) by studying the influence of language;

(vi) and, finally, by studying differences in CORE-OM scores according to subjects’ report of recent psychological stress.

## Methods

### Translation procedure

The translation into Norwegian was undertaken by Vidje Hansen (VH), an experienced psychiatrist and fluent in English. The Norwegian translation was then back-translated to English by a professional translator, and the two English versions were compared and discussed between VH and Chris Evans (CE), who is an experienced psychiatrist and a natural-born Englishman, and one of the creators of the CORE-OM. The agreed-upon Norwegian version was then tested out in a non-clinical and a clinical sample. Some minor adjustments were done, based upon respondents comments on readability of the items.

### Samples

A clinical sample (N=527) was collected from public out-patient mental health services in the counties of Finnmark (n=331), and the county of Nordland (n= 186), and from an out-patient university clinic at the Department of Psychology at the University of Tromsø (n=10). Altogether the clinical sample was constituted by 320 women and 207 men, with a mean age 37.4 (SD 12.6).

A non-clinical sample (N= 464) was collected from different sources: a convenience-sample recruited among family and friends of employees at out-patient mental health services in Finnmark county (n=182), a sample of medical students at the University of Tromsø (n=209), psychology students from the same university (n=61), and individuals attending a course aimed at passengers with fear of flying (n=12), altogether 333 women and 131 men. Mean age was 32.6 (SD 14.3).

A sub-sample of 81 medical and psychology students, fluent in both Norwegian and English language, participated in a “rotation experiment”. These bi-lingual students were assigned to four groups. These groups completed the CORE-OM in Norwegian and English language version, in different sequences (i.e. Norwegian-English-English-Norwegian; English-Norwegian-English-Norwegian, etc.), four times with one-week interval. The same sub-sample was also used for the for the test-retest reliability calculations.

### Consent

All sub-samples were informed in writing, according to the regulations laid down by the Regional Committee for Medical and Health Research Ethics in Health Region North (REC North) for this specific project, and return of the CORE-OM form was accepted as consent to participate in the project. All subjects were above 18 years of age.

### Ethics

The study was performed in compliance with the Helsinki Declaration for research on humans, and was approved by the REC North (Reference number 82/2004).

### Assessment

#### Demographic variables

Information collected about the participants was age and gender, and clinical versus non-clinical sample.

#### CORE-OM scoring

The CORE-OM paper and pencil version was used. The 34 items cover and yield scale scores from the four domains; Well-being (four items), Function (12 items), Problems/symptoms (12 items) and Risk (6 items). The time frame covered is the last week. Item-scores have minimum value of 0 and maximum value 4. Eight items are positively termed, and have reversed scoring. The total score (All) is calculated from the mean of all 34 items, and an alternative total score (All minus Risk) from the 28 non-risk items. All scores are calculated by dividing the sum item score by the number of items answered and multiplying the result by ten. The higher the score, the more troubled the patient is. For the 12 item subscales, scale scores were calculated based on the mean of the answered items (pro-rating) if no more than three items are missing; while for the two shorter sub-scales, only one item was allowed missing [4].

#### Measurement of psychological stress

The sub-sample of 81 students that participated in the test-retest reliability trial also answered a question meant to measure psychological stress, namely “Have you been exposed to any psychological stress last week?” Response categories were simply “Yes”, or “No”.

### Statistical analyses

#### Exploratory factor analysis (EFA)

The clinical sample was split in two parts using the first as an exploratory sample (Sample 1) and the second as a hold-out validation sample (Sample 2). A principal component analysis was conducted to extract principal components. As the CORE-OM is presumed to represent four domains, any range of one to four components was extracted. Promax rotation was chosen to allow for correlations among the component scores, which also made an analysis of second order factors possible. Component scores were saved using the regression method. The different EFA models were then compared in the validation sample using confirmatory factor analyses, and compared with the Four CORE domains model proposed by Evans and colleagues [4], and a general (*g*) factor model as described below.

#### Confirmatory factor analyses (CFA)

Seventeen of the 34 CORE-OM item scores were significantly skewed (*Z* ranging from −3.6 to 12.2; *M* = 1.7). Twenty-six items also indicated a non-normal kurtosis (*Z* ranging from −8.7 to +8.1), and hence, considerable multivariate kurtosis was present (Mardia’s = 48.8). As non-normal distributions bias estimation by narrowing the standard errors of the parameters, an asymptotic covariance matrix was estimated using PRELIS [15] and included as a weight matrix to adjust the error band. Polychoric correlations were calculated instead of Pearson correlations since ordinal scaling is a more realistic assumption than interval scaling in a four-point Likert scale. Robust maximum likelihood estimation was provided using Satorra-Bentler rescaled chi-square statistics (*SB χ*^*2*^). A non-significant chi-square statistic imply a perfectly fitting model, but as models almost always are not an exact replica of reality, root mean square error of approximation (RMSEA), standardized root mean error (SRMR) and comparative fit indices (CFI) were evaluated in addition to the *SB χ*^*2*^. Following Hu and Bentler [16], and Marsh and colleagues [17], RMSEA below < 0.06, SRMR < 0.08 and CFI above > 0.95 indicate a reasonably good model approximation.

The non-nested factor models (the four EFA models and the Four CORE domains model) were compared by putting most weight on the SB chi-square statistics and on the Akaike’s information criterion (AIC), which indicates the best combination of model fit and parsimony [17]. AIC was calculated as follows: *AIC* = *SBχ*^2^ + 2*p*  (*p* = number of model parameters). The AIC hence penalizes more advanced models. A lower AIC indicates a better fit. The *g*-factor model represents a nested factor model as the CORE item variances are modelled as a product of three latent variance components: a general factor (*g*) explaining the variance across all items, one or several specific factors explaining covariance patterns between the CORE items (according to the EFA), and an error component. A similar factor modelling approach has previously been conducted for the CORE by Lyne and colleagues [9], which explicate the modelling strategy.

#### Calculations of test parameters

Face validity of a questionnaire is best measured by acceptability, the percentage of items left unanswered. In the British validation [8] the overall omission rate was 1.7% of all items; while in the Swedish validation a mean omission rate of 0.44% was found.

SPSS version 18 was used for calculating basic statistics, differences between groups, linear regression modelling and test-retest reliability. For calculating a weighted average cut-off point, the formula of Jacobson and Truax [18] was employed:

$$\frac{M_{\text{clin}}SD_{\text{norm}}+M_{\text{norm}}SD_{\text{clin}}}{SD_{\text{clin}}+SD_{\text{norm}}}$$

## Results

### Acceptability

In the non-clinical sample 92% completed all 34 items. There were no single items that were uncompleted by more than 1.7% of the sample. Using pro-rating for persons with up to 3 items missing, 98.9% of the sample was usable for a sum-score. In the clinical sample, 83.1% completed all items, and no items were uncompleted by more than 3.6% of the sample (i.e. 97.5% usable).

### Domain score correlations

Two separate Pearson’s product moment correlation matrixes for the sums of scores on the four CORE domain sub-scales, one for the non-clinical and one for the clinical sample are presented in Table 1. Only the clinical sample was used for the principal components and confirmatory factor analyses.

**Table 1:** Inter-correlations (Pearson’s r) between CORE sub-scales in non-clinical and clinical samples

	**Functioning**	**Risk**	**Well-being**
Non-clinical (n=460)			
Symptoms/Problems	0.66**	0.37**	0.68**
Functioning		0.34**	0.71**
Risk			0.29**
Clinical (n=519)			
Symptoms/Problems	0.72**	0.63**	0.74**
Functioning		0.62**	0.68**
Risk			0.58**

** p<0.001.

### Exploratory factor analyses (EFA)

Thirteen cases had to be removed due to more than 10% missing data. Remaining missing values were replaced by multiple imputations using the expected maximum function in PRELIS. According to Kaiser’s criterion (eigenvalues > 1), seven principal components could be extracted. However, as the three last components (five to seven) contained few items (three or less), a model of four components represented the best combination of model fit (*R*^*2*^ = 51.7%, i.e. explained variance) and parsimoniousness (four components). The component loadings are presented in Table 2. The psychological distress items loaded on the first principal component. Seven out of eight positively phrased items, most of them from the Functioning domain, loaded on the second component. The items that loaded on the third component concerned dysfunctional relationships and self-blame. The fourth component included items concerning violence, threats and irritability towards others.

**Table 2:** **Principal component analysis in clinical sample 1 (*n *= 257)**

	**Principal components**			
CORE item # (sub-scale)^1^	1	2	3	4
5 (P) I have felt totally lacking in energy and enthusiasm	.84			
15 (P) I have felt panic or terror	.70			
11 (P) Tension and anxiety have prevented me doing important things	.67			
2 (P) I have felt tense, anxious or nervous	.66			
8 (P) I have been troubled by aches, pains or other physical problems	.65			
18 (P) I have had difficulty getting to sleep or staying asleep	.63			
17 (W) I have felt overwhelmed by my problems	.62			
20 (P) My problems have been impossible to put to one side	.60			
10 (F) Talking to people has felt too much for me	.58			
23 (P) I have felt despairing or hopeless	.58	.35		
14 (W) I have felt like crying	.54			
13 (P) I have been disturbed by unwanted thoughts or feelings	.53			
21 (F) I have been able to do most things I needed to	.49	.35		
19 (F) I have felt warmth and affection for someone	-.39	.79		
31 (W) I have felt optimistic about my future		.70		
9 (R) I have thought of hurting myself		.64		
4 (W) I have felt OK about myself		.63		
24 (R) I have thought it would be better if I were dead		.62		
12 (F) I have been happy with the things I have done		.61		
16 (R) I made plans to end my life		.57		.31
3 (F) I have felt I have someone to turn to for support when needed	-.33	.53	.32	
32 (F) I have achieved the things I wanted to		.52		
27 (P) I have felt unhappy	.33	.46		
7 (F) I have felt able to cope when things go wrong		.42		
1 (F) I have felt terribly alone and isolated		.39	.33	
25 (F) I have felt criticised by other people			.77	
33 (F) I have felt humiliated or shamed by other people			.74	
30 (P) I have thought I am to blame for my problems and difficulties		.36	.61	
26 (F) I have thought I have no friends			.55	
28 (P) Unwanted images or memories have been distressing me	.33		.54	
6 (R) I have been physically violent to others				.77
22 (R) I have threatened or intimidated another person				.74
29 (F) I have been irritable when with other people	.35			.48
34 (R) I have hurt myself physically or taken dangerous risks with my health				.46
Eigenvalues	12.2	1.9	1.8	1.7
Explained variance (%)	35.8	5.6	5.4	4.9

*Note*. Total variance explained = 51.7 %. Component loadings < .30 are suppressed.

^1^Domain names: W = Subjective well-being, P = Problems/symptoms, F = Functioning, R= Risk.

Saving the component scores and subjecting those to a second EFA, thus representing an analysis of second order components, revealed strong support for one general component (eigenvalue = 2.19) and weaker support of a second component (eigenvalue = .89). However, as the second component had a high loading (0.99), and represented the three risk items of the fourth component, this component provided a conceptually distinct additional independent contribution.

### Confirmatory factor analyses

**Figure 1:**
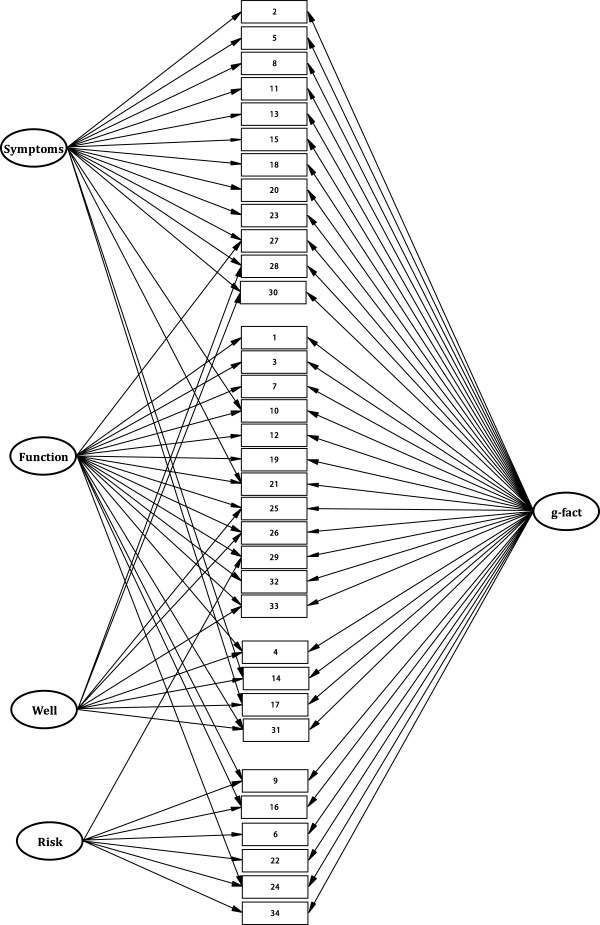
**Illustration of best fitting model**^**1 **^**in Confirmatory Factor Analysis. **^1^ Same as model 7 in Table 3 and the model presented in Table 4. Legend: Symptoms= Symptoms/ Problems, Function= Functioning, Well= Well-being, Risk= Risk, *g*-fact = *g*-factor. Numbers in squares are item numbers in the CORE-OM.

In the validation sample, several factor models were compared against each other, and against the null model (no factor loadings estimated). Again, the four-factor solution based on the EFA (model 4) from Table 2 received better support than the one, two or three component models (see Table 3). The model fit, according to the RMSEA index, was however not good (.0759). The Four CORE domains model as originally published by Evans (model 5) [4] was significantly poorer in absolute terms (worse *SB χ*^*2*^), but only slightly worse in terms of the RMSEA. However, specification of a general (*g*) factor (model 6) improved fit considerably both in absolute terms (considerable drop in chi-square and in AIC) and according to the RMSEA (.0605), now approaching a tenable model fit. By allowing factor side-loadings according to the original Four CORE domains model (model 7), model fit improved slightly more (RMSEA = .057). The comparative fit index (CFI) was also adequately high (.974). The loadings for the *g* and the specific factors of model 7 are presented in Table 4. This model is illustrated in Figure 1.

**Table 3:** **Comparisons of factor models using Structural Equation Modelling (SEM) in clinical sample 2 (*n *= 257)**

**Model**	***df***	***χ***^***2***^	***SB χ***^***2***^	***AIC***	***RMSEA***	***CFI***
0: Null model	561	15610.0	15010.0	15077.9	.3172	.040
1: One factor	527	2374.8	1778.4	1914.4	.0963	.917
2: Two factors	526	1963.7	1457.4	1595.4	.0831	.938
3: Three factors	524	1890.2	1360.8	1502.8	.0790	.944
4: Four factors	521	1784.7	1289.2	1437.2	.0759	.949
5: Four CORE domains	521	1854.7	1367.5	1515.5	.0797	.944
6: Four factors +g	493	1347.4	953.3	1157.3	.0605	.969
7: Four factors +g+ Four CORE domains^1^	477	1235.1	876.4	1112.4	.0572	.974

*Notes*. Factor scores were allowed to correlate in models 1–5. In model 6 factor scores are specified as independent, due to the general factor explaining factor covariance. ^1^ Same model as in Table 4 and Figure 1.

**Table 4:** **Results from a SEM analysis specifying a *g *factor and the four CORE domains in clinical sample 2 (*n *= 257)**

	**Common factors**				
**CORE items**	**P**^**1**^	**F**	**W**	**R**	***g***
2 (P) I have felt tense, anxious or nervous	.50				.58
5 (P) I have felt totally lacking in energy and enthusiasm	.47				.43
8 (P) I have been troubled by aches, pains or other physical problems	.35				.20
11 (P) Tension and anxiety have prevented me doing important things	.41				.50
13 (P) I have been disturbed by unwanted thoughts or feelings	.28				.58
15 (P) I have felt panic or terror	.52				.41
18 (P) I have had difficulty getting to sleep or staying asleep	.22				.39
20 (P) My problems have been impossible to put to one side	.38				.36
23 (P) I have felt despairing or hopeless	.25				.74
27 (P) I have felt unhappy	.26	(−.03)			.75
28 (P) Unwanted images or memories have been distressing me	.20		(.17)		.49
30 (P) I have thought I am to blame for my problems and difficulties	.01		(.24)		.50
*R*^*2*^	.12	.00	.01	.00	.27
1 (F) I have felt terribly alone and isolated		.13			.47
3 (F) I have felt I have someone to turn to for support when needed		.19			.37
7 (F) I have felt able to cope when things go wrong		.26			.69
10 (F) Talking to people has felt too much for me	(.40)	.21			.39
12 (F) I have been happy with the things I have done		.39			.69
19 (F) I have felt warmth and affection for someone		.14			.31
21 (F) I have been able to do most things I needed to	(.08)	.20			.49
25 (F) I have felt criticized by other people		.19	(.60)		.40
26 (F) I have thought I have no friends		.13	(.39)		.43
29 (F) I have been irritable when with other people		.31		(.35)	.18
32 (F) I have achieved the things I wanted to		.19			.63
33 (F) I have felt humiliated or shamed by other people		.14	(.50)		.40
*R*^*2*^	.01	.05	.06	.01	.23
4 (W) I have felt OK about myself		(.45)	-.11		.64
14 (W) I have felt like crying	(.21)		-.01		.48
17 (W) I have felt overwhelmed by my problems	(.51)		-.04		.60
31 (W) I have felt optimistic about my future		(.16)	-.19		.59
*R*^*2*^	.08	.06	.01	.00	.34
6 (R) I have been physically violent to others				.69	.36
9 (R) I have thought of hurting myself		(−.31)		.06	.82
16 (R) I made plans to end my life		(−.39)		.08	.81
22 (R) I have threatened or intimidated another person				.76	.32
24 (R) I have thought it would be better if I were dead		(−.19)		.05	.85
34 (R) I have hurt myself physically or taken dangerous risks with my health				.49	.59
*R*^*2*^	.00	.05	.00	.22	.44

*Notes*. *R*^*2*^ = Explained variance (%). Factor loadings in parentheses were estimated as specified by the principal component analysis (from Table 2).

^1^ Domain names: P =Symptoms/ Problems, W= Subjective well-being, F = Functioning, Risk=Risk, *g* = General factor.

Finally, it was examined to what extent methodical aspects affected the item responses, similarly as Lyne and colleagues [9] examined. Two additional factors were specified, for the positively and negatively framed items, respectively. The loadings for each method factor were constrained equal under the assumption that a method effect should exert a relatively equal influence on all items, but also in order to maximize degrees of freedom given the medium sample size. Model fit did not improve significantly (*S-B* χ^2^ = 5.27, *p* = .07). The changes in the RMSEA and the CFI indices were barely observable and the factor loadings were small, .08 for the positively and .12 for the negatively framed items, hence explaining about 1.2% of the variance. Control of response bias was thus of less concern and dropped from further interpretation.

### Interpretation of factor loadings

The *g* factor (Table 4 and Figure 1) is the most important dimension, as it explains most of the variance in the CORE items. For the items assessing the ‘Problem/Symptoms’ factor, two-thirds of the variance was explained by the *g*, and one-third by its specific factor. Two-thirds of the variance in the items assessing functioning, was also explained by the *g*, while 14 and 17 percent were explained by the specific factors ‘Functioning’ and ‘Well-being’, respectively. For the ‘Well-being’ items, however, two-thirds was explained by *g*, and the remaining variance was partly explained by the specific ‘Problems/Symptoms’ and ‘Functioning’ factors. Thus, it was not possible to validate the CORE subjective well-being domain. However, there seems to be a common pattern of low self-regard and interpersonal problems in the items from the Problems/Symptoms and Functioning domain that loads on the “well-being” factor.

About 62 percent of the variance in the risk items was explained by the *g* factor. However, three of the risk items, which are inherent in internalising problems, signalling self-harm and thoughts of suicide (e.g., thoughts of hurting myself, suicidal ideation, or making plans for suicide) were most strongly explained by *g*, while the three externalizing, or acting out, risk items (e.g., hurt myself physically or taken dangerous risks, being physically violent, and threatening against other people), were most strongly explained by the specific ‘Risk’ factor. This demonstrates that the classical suicidal and self-harm risk items are important indicators of general psychological distress, while the three acting out risk items should be assessed independently of the sum or *g* score. This interpretation is in line with the second order EFA above, which supported interpreting the fourth component (representing the same three risk items) as independent from the other component.

### Internal consistency

The internal consistency of the CORE-OM is presented in Table 5. For both total scores and separate domains consistency was generally high. The lowest internal consistency was found for the risk domain in the non-clinical sample, while the α of the risk domain was significantly higher in the clinical sample.

**Table 5:** Internal consistency, Chronbach’s α (95% CI), for non-clinical and clinical samples

**Domain**	**Non-clinical (n=473)**	**Clinical (n=528)**
Subjective well-being (4 items)	0.74 (0.70-0.78)	0.70 (0.66-0.74)
Symptoms / Problems (12 items)	0.84 (0.82-0.86)	0.87 (0.85-0.88)
Functioning (12 items)	0.82 (0.80-0.85)	0.84 (0.81-0.86)
Risk (6 items)	0.68 (0.63-0.72)*	0.81 (0.78-0.83)*
Non-risk items (28 items)	0.92 (0.91-0.93)	0.93 (0.92-0.94)
All items (34 items)	0.92 (0.91-0.93)	0.94 (0.93-0.95)

*p<0.05 (significantly higher α in clinical sample).

### Test-retest reliability, and effect of Norwegian versus English language versions

For test-retest reliability calculations, stability of the same language versions of the CORE (Norwegian-Norwegian), filled in one or two weeks apart by the 81 students participating in the language experiment was used. In Table 6, test-retest stability of domain and total scores for the Norwegian language version is shown. For all domains, except the risk domain, the stability was moderately high. Test-retest stability for the language rotation experiment was also studied. By Pearson’s r, test-retest reliability in the language rotation experiment ranged between 0.50 to 0.87 for the All items score of Norwegian version, and between 0.74 and 0.87 for the English version. In multiple regression analyses, with sex, age, language, and psychological stress as predictors, language was only a significant predictor of total CORE score at the fourth filling-in (β=0.27, p=0.01). On all other occasions, the effect of language was non-significant.

**Table 6:** Test-retest stability in non-clinical sample of 81 students

	**Well-being**	**Symptoms/ Problems**	**Functioning**	**Risk**	**Non-risk items**	**All items**
Well-being	0.63^1^*					
Symptoms/ Problems		0.69*				
Functioning			0.70*			
Risk				0.35*		
Non-risk items					0.76*	
All items						0.76*

^1^ Correlations between domain scores (Spearman’s rho).

*p<0.01.

### Gender and age differences

Table 7 shows the mean and standard deviations for the four domain-scores, for the non-risk items, and for the total (All items). With only two exceptions, there were no significant differences in mean-scores between the sexes, neither in the non-clinical nor in the clinical sample. In the non-clinical sample the mean score was significantly higher in women. The score for risk in the clinical sample was significantly higher in men.

**Table 7:** Mean (M) and gender differences in scores for non-clinical and clinical sample

**Domain**	**Non-clinical**							**Clinical**						
	**Male**		**Female**			**Total**		**Male**		**Female**			**Total**	
	**n=130**		**n=330**			**N=440**		**n=206**		**n=313**			**n=529**	
	**M**	**SD**	**M**	**SD**	**95 % CI**^**1**^	**M**^**2**^	**SD**	**M**	**SD**	**M**	**SD**	**95 % CI**^**1**^	**M**^**2**^	**SD**
Well-being	0.74	0.60	0.98	0.67	0.11 – 0.37^*^	0.92	0.66	2.16	0.79	2.29	0.79	−0.00 – 0.80	2.25^**^	0.79
Symptoms	0.76	0.54	0.85	0.57	−0.02 – 0.21	0.83	0.56	2.25	0.79	2.25	0.75	−0.12 – 0.15	2.26^**^	0.77
Functioning	0.73	0.50	0.76	0.45	−0.07 – 0.12	0.76	0.47	1.75	0.66	1.69	0.64	−0.18 – 0.06	1.71^**^	0.65
Risk	0.09	0.23	0.06	0.19	−0.07 – 0.01	0.07	0.20	0.76	0.81	0.52	0.63	−0.37 – 0.13^*^	0.62^**^	0.71
Non-risk	0.74	0.48	0.83	0.49	−0.07 – 0.19	0.81	0.49	2.03	0.68	2.01	0.64	−0.12 – 0.11	2.03^**^	0.66
All items	0.60	0.42	0.69	0.41	−0.03 – 0.16	0.68	0.42	1.80	0.66	1.75	0.60	−0.16 – 0.06	1.78^**^	0.63

^1^ 95% confidence interval for difference between male and female scores.

^2^ Means in non-clinical and clinical samples compared by t-test, significance of differences reported in M column for clinical sample.

^*^p<.001, ^**^p<.0001.

All mean scores were significantly higher in the clinical than in the non-clinical sample.

To check whether differences between clinical and non-clinical samples were influenced by the differences in age and gender distribution in the two samples, stepwise linear regression modelling was performed. The results are presented in Table 8.

**Table 8:** Stepwise linear regression analyses for CORE scores and domains, controlling for gender, age and clinical status

	**Dependent variable in model**											
**Independent variables**^**3**^	**Well-being**^**1**^		**Symptoms**		**Functioning**		**Risk**		**Non-risk**		**All items**	
	**β**^**2**^	**p<**	**β**	**p<**	**β**	**p<**	**β**	**p**	**β**	**p**	**β**	**p**
Gender	0.08	.001	0.02	n.s.	−0.02	n.s.	−0.14	.0001	0.02	n.s.	0.00	n.s.
(Male=0, female=1)												
Age	−0.04	n.s.	0.00	n.s.	−0.06	.05	−0.11	.0001	−0.30	n.s.	−0.04	n.s.
Patient status	0.69	.0001	0.73	.0001	0.65	.0001	0.46	.0001	0.73	.0001	0.72	.0001
(Non-clinical=0, Clinical=1)												

^1^ Model fit statistics (ANOVA) with df=3 for all models: Well-being: F=279.1, p<.0001; Symptoms/ Problems: F=363.7, p<.0001; Functioning: F=232.3, p<.0001; Risk: F= 98.6, p<.0001; Non-risk: F= 352.1, p<.0001; All items: F= 338.0, p<.0001.

^2^ β Standarized Beta of independent variables.

^3^ Variables entered stepwise in order: Gender + Age+ Patient status.

The highest proportion of the variance for all CORE scores, both sub-domain and sum scores, was accounted for by current patient status (i.e. belonging to the Clinical sample). In addition to current patient status, younger age accounted for part of the variance in Functioning and Risk. Furthermore, being a woman predicted worse Well-being scores, and being a man predicted higher Risk scores. However the difference between the two samples was mainly accounted for by current patient status, while age and gender distribution had some, but negligible effects.

### Clinical Cut-off scores

The cut-off scores for clinical significant change are presented in Table 9. Women had higher scores on well-being (i.e.: feeling less well). Otherwise cut-off scores were fairly equal for men and women.

**Table 9:** Cut-off scores between clinical and non-clinical samples in men and women

	**Men**	**Women**
Well-being	1.34	1.58
Symptoms / Problems	1.35	1.46
Functioning	1.16	1.14
Risk	0. 22	0.28
Non-risk items	1.26	1.34
All items	1.08	1.12

### The influence of psychological stress

To further study the validity of the cut-off scores for clinical significant change, the sub-sample of 81 students that participated in the four week language rotation experiment were each week asked “Have you been exposed to any psychological stress last week?”. For all domains, in the first week of the experiment, the CORE-OM scores differed according to whether the subjects reported experiencing stress (Table 10). Those who experienced stress had a total CORE-OM score (All items) almost exactly on the clinical cut-off point.

**Table 10:** Difference in CORE scores (ANOVA) between students who reported experiencing psychological stress, or no stress last week (N=81)

						**Anova**	
**Domain**	**Stress**	**N**	**Mean**	**SD**	**95% C I for Mean**	**F**	**p**
Well-being	Stress	13	1.28	0.44	1.02-1.56	14.124	0.000
	No stress	68	0.68	0.55	0.54-0.81		
Symptoms/Problems	Stress	12	1.16	0.43	0.88-1.43	8.170	0.005
	No stress	68	0.75	0.46	0.64-0.86		
Functioning	Stress	13	1.09	0.39	0.86-1.33	17.571	0.000
	No stress	68	0.64	0.35	0.56-0.73		
Risk	Stress	13	0.30	0.51	−0.01-0.60	10.526	0.002
	No stress	68	0.05	0.16	0.01-0.09		
Non-risk items	Stress	13	1.17	0.36	0.95-1.39	19.950	0.000
	No stress	68	0.69	0.39	0.60-0.79		
All items	Stress	13	1.02	0.36	0.80-1.23	18.665	0.000
	No stress	68	0.58	0.33	0.50-0.66		

**Figure 2:**
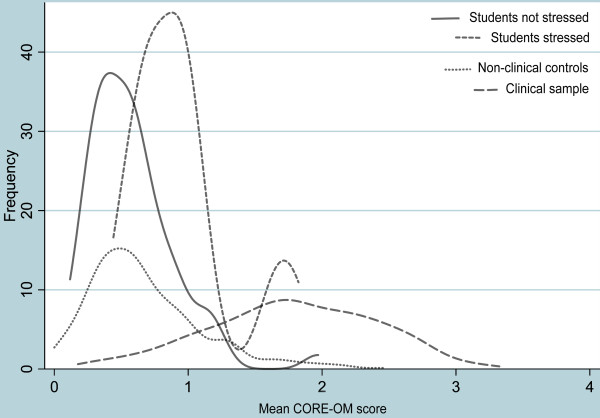
Distributions of CORE-OM (All items) scores in clinical and non-clinical samples, and in students reporting stress, or no stress, during last week.

The distributions of CORE-OM All items scores are illustrated in Figure 2 showing the distributions of the Clinical and Non-clinical total samples, and of the students reporting psychological stress or not during the preceding week.

As the figure illustrates, the clinical sample has a wide distribution, covering the whole spectrum of CORE-scores, with the vast majority of cases well above 1.0. On the other hand, the non-clinical sample has a wide and somewhat skewed distribution, with a thin “arm” reaching well into the clinical population. However the vast majority of the non-clinical sample has CORE-scores well below 1.0. The student sample was separated by the “stress”-item. Students reporting no stress were almost all well below clinical cut-off, while students reporting stress formed a bimodal distribution, the larger peaking close to CORE-score 1.0, while the smaller peak coincided with the mode of the clinical sample. The Norwegian translation is presented alongside the English original CORE-OM in Table 11. The items are ordered according to the originally proposed domains and sub-domains.

**Table 11:** The CORE-OM items in English and Norwegian translation, sorted after domain and sub-domains, and with item number

**Domain**	**Item**	**English original**	**Norwegian translation**
		**Over the last week…**	**I løpet av den siste uken…**
W	4^1^	I have felt OK about myself	Har jeg følt meg fornøyd med meg selv
W	14	I have felt like crying	Har jeg hatt lyst til å gråte
W	17	I have felt overwhelmed by my problems	Har jeg følt meg overveldet av mine problemer
W	31^1^	I have felt optimistic about my future	Har jeg følt meg optimistisk med tanke på framtiden
P-anxiety	2	I have felt tense, anxious or nervous	Har jeg følt meg anspent, engstelig eller nervøs
P-anxiety	11	Tension and anxiety have prevented me doing important things	Har anspenthet og angst hindret meg i å gjøre viktige ting
P-anxiety	15	I have felt panic or terror	Har jeg følt redsel eller panikk
P-anxiety	20	My problems have been impossible to put to one side	Har det vært umulig å legge bort problemene mine
P-depressed	5	I have felt totally lacking in energy and enthusiasm	Har jeg følt meg helt uten energi og entusiasme
P-depressed	23	I have felt despairing or hopeless	Har jeg følt meg fortvilet eller uten håp
P-depressed	27	I have felt unhappy	Har jeg følt meg ulykkelig
P-depressed	30	I have thought I am to blame for my problems and difficulties	Har jeg tenkt at mine problemer eller vanskeligheter var min egen skyld
P-physical	8	I have been troubled by aches, pains or other physical problems	Har jeg vært plaget av verk, smerter eller andre fysiske plager
P-physical	18	I have had difficulty getting to sleep or staying asleep	Har jeg hatt problemer med å sovne eller har våknet fort igjen
P-trauma	13	I have been disturbed by unwanted thoughts or feelings	Har jeg vært plaget av uønskede tanker eller følelser
P-trauma	28	Unwanted images or memories have been distressing me	Har uønskede bilder eller minner plaget meg
F-general	7^1^	I have felt able to cope when things go wrong	Har jeg følt meg i stand til å takle det når noe har gått galt
F-general	12^1^	I have been happy with the things I have done	Har jeg vært fornøyd med det jeg har gjort
F-general	21^1^	I have been able to do most things I needed to	Har jeg klart å gjøre det meste av det jeg hadde behov for å gjøre
F-general	32^1^	I have achieved the things I wanted to	Har jeg fått til det jeg ville
F-close	1	I have felt terribly alone and isolated	Har jeg følt meg forferdelig alene og isolert
F-close	3^1^	I have felt I have someone to turn to for support when needed	Har jeg følt at jeg hadde noen å støtte meg til når jeg trengte det
F-close	19^1^	I have felt warmth and affection for someone	Har jeg følt varme og hengivenhet overfor noen
F-close	26	I have thought I have no friends	Har jeg tenkt at jeg ikke hadde noen venner
F-social	10	Talking to people has felt too much for me	Har det å snakke med folk vært for mye for meg
F-social	25	I have felt criticised by other people	Har jeg følt meg kritisert av andre
F-social	29	I have been irritable when with other people	Har jeg vært irritabel mot andre mennesker
F-social	33	I have felt humiliated or shamed by other people	Har jeg følt at andre har ydmyket meg eller gjort meg skamfull
R-self	9	I have thought of hurting myself	Har jeg tenkt på å skade meg selv.
R-self	34	I have hurt myself physically or taken dangerous risks with my health	Har jeg skadet meg selv fysisk eller tatt farlige sjanser med min egen helse
R-suicidal	16	I made plans to end my life	Har jeg lagt planer for å gjøre slutt på livet mitt
R-suicidal	24	I have thought it would be better if I were dead	Har jeg tenkt det ville vært bedre om jeg var død
R-others	6	I have been physically violent to others	Har jeg vært fysisk voldelig mot andre
R-others	22	I have threatened or intimidated another person	Har jeg truet eller skremt et annet menneske

W= Well-being; P= Symptoms/ Problems; F= Functioning; R= Risk; ^1^ Reversed scoring.

## Discussion

The main finding of this study was that the CORE-OM in Norwegian translation can be seen as an instrument measuring a *g* factor of psychological distress and simultaneously confirming subordinated factors measuring problems, functioning and risk domains. The content of the original last theorized domain, well-being, could not be confirmed in our data. On the other hand, a domain concerning low self esteem and interpersonal problems emerged. Our findings also support simplifying the communication of CORE-scores into two: Psychological distress and Risk.

Furthermore we found that both the internal consistency and the test-retest stability of the CORE-OM were acceptable, and compared well with the original English normative data. We also found that language version had no or only negligible influence on CORE-scores in a bilingual student sample. Finally, the clinical cut-off points in the Norwegian samples were fairly equal to the English norms. The proposed clinical cut-off point of 1.0 was further validated, by demonstrating that subjects reporting no psychological stress were separated from those reporting psychological stress. Thus, our findings did not support that CORE-OM fully measures the domains suggested by the phase model of psychotherapy [4,6]. The original Four CORE domains model as proposed by Evans [4] received poor support, due to several items loading on other factors than theoretically intended. As can be seen from the results of the EFA (principal components analysis), and the confirmatory factor analyses, when controlling for the *g* factor, the originally proposed *problem/symptom* items that loaded on the *problems/symptoms* component related to anxiety and distress. Furthermore, the originally proposed *functioning* items that still retained some variance in a secondary *functioning* factor related to everyday coping and lack of positive affect. Interestingly, the original *well-being* domain was not confirmed in our data, and instead an *interpersonal problems* factor emerged. Finally the *risk* domain separated into *risk to self* and *risk to others*, of which the *risk to self* -items loaded strongly on the *g* factor of psychological distress, while the *risk to others* items loaded most strongly on a separate *risk* factor. The more ambiguous risk item, concerning risk taking, loaded almost equally on the *g* and the *risk* factors.

Interestingly, the *g* factor, as well as the *problems*, *functioning* and *risk* factors correspond well with the “state” concept of psychopathology [11]. On the other hand, the items clustering around the *interpersonal relations* theme are more in line with a trait concept, since the content of these items could be attributed to stable problems of personality [11]. However, contrary to Bedford and colleagues [10], we do not find this seeming mixture of state and trait items in the CORE-OM problematic. Personality traits are unlikely to change much in psychotherapy, and could be regarded redundant in an outcome measure. On the other hand, endorsement of the same items could indicate that the patient is living in a pathogenic relationship or environment. Since the CORE-OM is intended to be a general clinical outcome instrument, which can give the clinician useful hints about the problem profile of the patient, scoring on the low self esteem and problematic interpersonal relation items should lead to exploration of depression, personality problems, living conditions, interpersonal relations, and trauma history. Thus our recommendation to clinicians is to give attention not only to total and to separate domains, but also to separate items scores when using CORE-OM.

However, since the variance of the majority of the CORE items was most strongly related to the *g* factor, the creation of a single sum score to assess symptom load appears adequate for effective clinical communication, rather than creating four separate subscale sum scores, which are highly correlated, as well. Also, the risk items should be consulted independently. Our findings were in accordance with Lyne and colleague’s [9], who proposed that CORE-scores should be communicated through two scores: *General psychological distress* and *risk*.

In the present validation study of the Norwegian CORE-OM, we studied psychometric properties as acceptability, internal consistency, test-retest stability, and the differences between scores in non-clinical and clinical samples. These properties were considered comparable with the results from validation studies in England [8,14], Sweden [7], Italy and Japan [12,13]. The present cut-off scores were also in line with previous results. Connell and colleagues [14] recommended a cut-off score of 1.0 for the total score irrespective of gender. They argue that since psychological distress is a matter of degree, and not a discrete phenomenon, any cut-off score will to some degree be arbitrary. Interestingly, no substantial gender differences were found in our clinical data, other than for the higher risk scores in men. Correspondingly we can assume that men and women when psychologically distressed experience much the same load of problems.

Our data, comparing the scores of students reporting psychological stress to those reporting no stress, confirms that 1.0 is a valid cut-off point separating those who are in distress from those who are relatively unaffected by psychological problems. Based on our data, the cut-off scores for the CORE-OM in Norway correspond to the English norms.

### Strengths and limitations

The strengths of this study were the large sample of out-patients from Norwegian mental health services, the confirmatory factor analysis, the alternate language versions experiment, and the measurement of psychological stress. Some possible limitations should however be noted.

The non-clinical sample was collected among friends of employees at out-patient clinics, and among students of medicine and psychology. This means that the sample is probably not representative of the Norwegian population. The sample most probably consists of persons with a somewhat higher social status than the population, and as such, probably with better mental health.

Differences in age and gender distribution between the clinical and non-clinical samples could confound both the differences between the two samples and the calculated cut-off points for clinical significant change. Regression analysis did however demonstrate that even though age and gender did contribute to the variance for some sub-scores, currently being a patient was the strongest predictor of differences between members of the two samples.

The language rotation experiment resulted in finding that language version did affect the scores on the fourth and last occasions of filling in the questionnaire. Thus our findings did not rule out the possibility of language or translation having influenced the results.

Although a measurement of psychological stress was introduced in the sample of students, no specification of the nature of stress was obtained.

No convergent validation towards other clinical measures was performed. Since our sample was collected anonymously from different populations, we did not have data for cross-validation. However, the CORE-OM in Swedish, English, Italian and Japanese has been cross-validated with other measures like the BDI, BAI, GHQ and SCL [7,8,12,13], and another cross-validation seems redundant.

## Conclusions

The Norwegian translation of the CORE-OM have psychometric properties at the same high level as the English original, and could be recommended for general clinical use in out-patient populations. The present study provided most support for a general factor (g) underlying the CORE-OM items, while the sub-domain factors were less distinctively defined. Moreover, the risk items for harm should be consulted separately. For easy clinical communication we recommend that both the total CORE-OM scores and the risk scores should be flagged.

## Competing interests

The authors declare that they have no competing interests.

## Authors’ contributions

IS, VH and CE contributed to the conception and design of the study. VH, IS, LM, SE, KL and KS contributed to the acquisition of data. VH, IS and OF contributed to the analysis of data and interpretation of results. IS, VH and OF drafted the manuscript. LM, SE, KL and KS were involved in the critical revision of the manuscript. All authors read and approved the final version.

## Pre-publication history

The pre-publication history for this paper can be accessed here:

http://www.biomedcentral.com/1471-244X/13/99/prepub
